# Binding Affinity Determines Substrate Specificity and Enables Discovery of Substrates for N-Myristoyltransferases

**DOI:** 10.1021/acscatal.1c03330

**Published:** 2021-11-29

**Authors:** Dan Su, Tatsiana Kosciuk, Min Yang, Ian R. Price, Hening Lin

**Affiliations:** † Department of Chemistry and Chemical Biology, Cornell University, Ithaca, New York 14853, United States; ‡ Howard Hughes Medical Institute; Department of Chemistry and Chemical Biology, Cornell University, Ithaca, New York 14853, United States

**Keywords:** N-myristoyltransferase, substrate specificity, enzymes, post-translational modifications, Michaelis−Menten, binding affinity, *k*_cat_/*K*_m_

## Abstract

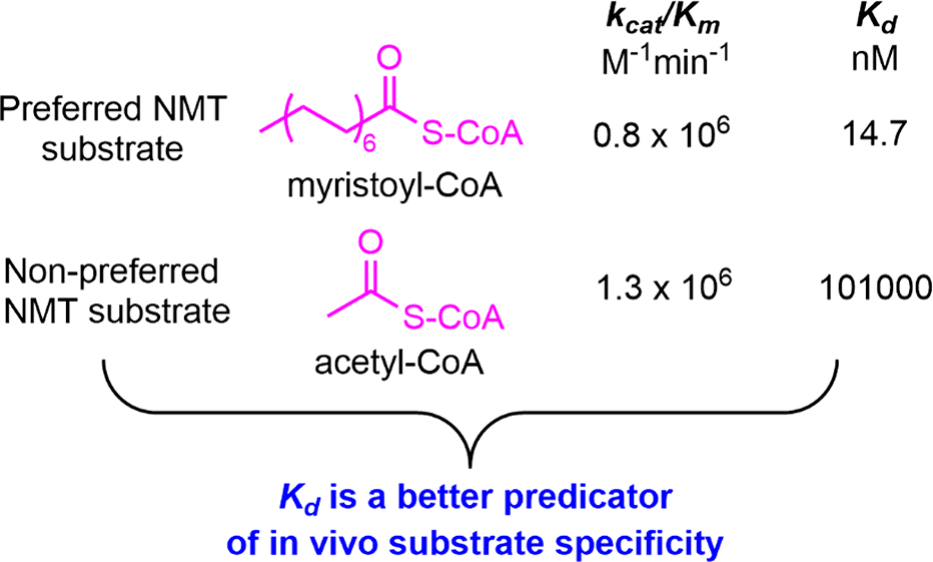

Kinetic parameters (*k*_cat_ and *K*_m_) derived from the Michaelis–Menten equation are widely used to characterize enzymes. *k*_cat_/*K*_m_ is considered the catalytic efficiency or substrate specificity of an enzyme toward its substrate. N-Myristoyltransferases (NMTs) catalyze the N-terminal glycine myristoylation of numerous eukaryotic proteins. Surprisingly, we find that in vitro human NMT1 can accept acetyl-CoA and catalyze acetylation with *k*_cat_ and *K*_m_ values similar to that of myristoylation. However, when both acetyl-CoA and myristoyl-CoA are present in the reaction, NMT1 catalyzes almost exclusively myristoylation. This phenomenon is caused by the dramatically different binding affinities of NMT1 for myristoyl-CoA and acetyl-CoA (estimated *K*_d_ of 14.7 nM and 10.1 μM, respectively). When both are present, NMT1 is essentially entirely bound by myristoyl-CoA and thus catalyzes myristoylation exclusively. The NMT1 example highlights the crucial role of binding affinity in determining the substrate specificity of enzymes, which in contrast to the traditionally held view in enzymology that the substrate specificity is defined by *k*_cat_/*K*_m_ values. This understanding readily explains the vast biological literature showing the coimmunoprecipitation of enzyme–substrate pairs for enzymes that catalyzes protein post-translational modifications (PTM), including phosphorylation, acetylation, and ubiquitination. Furthermore, this understanding allows the discovery of substrate proteins by identifying the interacting proteins of PTM enzymes, which we demonstrate by identifying three previously unknown substrate proteins (LRATD1, LRATD2, and ERICH5) of human NMT1/2 by mining available interactome data.

The Michaelis–Menten equation is the best-known model for enzymatic reactions described in biochemistry textbooks. Steady-state kinetic parameters derived from the Michaelis–Menten equation and measured experimentally, the *k*_cat_ and *K*_m_ values, are widely used to characterize enzymatic reactions. Specifically, *k*_cat_ is a term that defines the maximal rate, *K*_m_ is the substrate concentration at which reaction reaches half of its maximal rate, and *k*_cat_/*K*_m_ is considered a measure of the catalytic efficiency or substrate specificity.^1^ A substrate with a higher *k*_cat_/*K*_m_ value is considered a better or preferred substrate. While studying human N-myristoyltransferase 1 (NMT1), we unexpectedly discovered that NMT1 shows a drastically different preference for two substrates with similar *k*_cat_/*K*_m_ values, leading to the turnover of only one of the substrates when both were present in the reaction. This observation indicates that binding affinity is a more effective predictor of substrate specificity than *k*_cat_/*K*_m_. Our findings have important biological and medicinal implications warranting additional considerations for enzyme substrate and inhibitor discovery. Furthermore, we demonstrate that binding affinity-based approaches allow a facile identification of substrate proteins for enzymes that control protein post-translational modifications (PTM).

## Results and Discussion

### NMT1 Catalyzes Myristoylation and Acetylation of ARF6 Peptide with Similar *k*_cat_/*K*_m_ Values in Vitro

**Figure 1:**
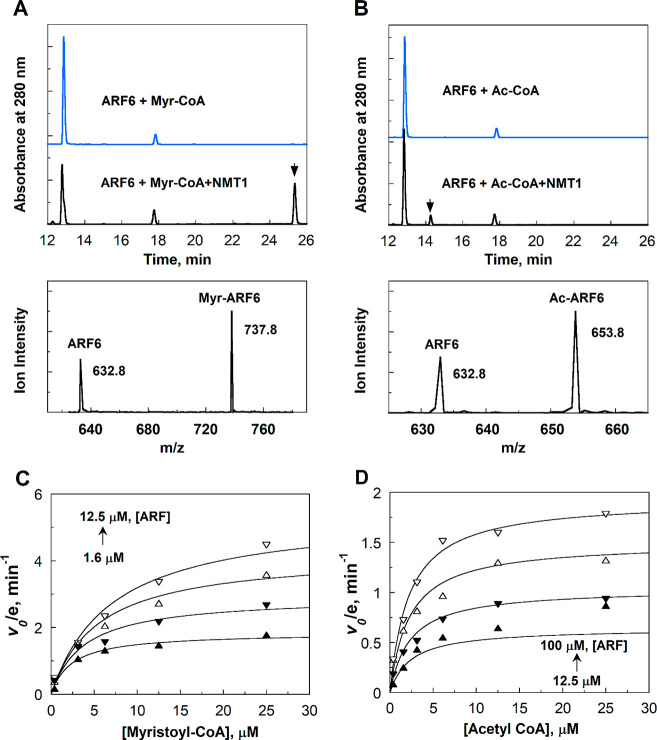
NMT1 catalyzes N-terminal myristoylation and acetylation on ARF peptide. (A) Free and myristoylated ARF6 peptides are detected by HPLC and mass spectrometry. (B) Free and acetylated ARF6 peptides are detected by HPLC and mass spectrometry. (C) Plot of initial rates of reaction versus varying concentrations of myristoyl-CoA with ARF6 peptide concentrations at 1.6 (▲), 3.1 (▼), 6.3 (Δ), and 12.5 (▽) μM. (D) Plot of initial rates of reaction versus varying concentrations of acetyl-CoA with ARF6 peptide concentrations at 12.5 (▲), 25 (▼), 50 (Δ), and 100 (▽) μM. Data were globally fit with eq 2 (see Material and Methods in the Supporting Information), and the derived steady-state kinetic parameters are summarized in Table 1.

N-terminal glycine myristoylation is an important and evolutionally conserved PTM in eukaryotic cells catalyzed by N-myristoyltransferases (NMTs).^2^ It regulates membrane targeting, protein stability, and protein–protein interactions of numerous human proteins essential to a broad spectrum of biological processes, including cancer progression, immune responses, and parasitic and viral infection.^3^ There are two NMTs in humans, NMT1 and NMT2, that are known to be very selective for myristoyl-CoA and N-terminal glycine of proteins. The preference for glycine is well understood from structural studies as the side chain of other amino acids are not tolerated in the active site because of steric reasons.^4^ However, recent studies demonstrated that human NMT1/2 can also catalyze the myristoylation of lysine 3 side chain of ARF6 protein.^5,6^ Previously, the activity of partially purified human NMT was tested on a variety of acyl-CoA molecules ranging from C7 to C16, and myristoyl-CoA is shown to be the most preferred substrate.^7^ However, shorter acyl-CoA molecules were not tested. Surprisingly, when we tested recombinant human NMT1 with ARF6 N-terminal peptide (GKVLSKIFWW), we found that acetyl-CoA can also be used as a substrate, leading to the formation of an acetyl peptide product (Figure 1). As expected, a new peak with a retention time of ∼25 min was observed in the HPLC chromatogram for the reaction containing ARF6 peptide, myristoyl-CoA, and NMT1, compared with the corresponding control reaction without NMT1. The identity of the myristoylated ARF6 peptide was further confirmed by mass spectrometry (*m*/*z* = 737.8, doubly charged) (Figure 1A). Unexpectedly, in the reaction containing ARF6 peptide, acetyl-CoA, and NMT1, the production of acetylated ARF6 peptide was also observed (retention time ∼14 min on HPLC and *m*/*z* = 653.8 doubly charged on MS) (Figure 1 B). The lysine of this peptide was free of myristoylation and acetylation as previously reported.^5^

Because it is well-known that NMT1 selectively uses myristoyl-CoA as a substrate, the observation that it can catalyze acetylation in vitro was surprising. We therefore hypothesized that in vivo, NMT1 could still predominately catalyze myrisotylation as long as the *k*_cat_*/K*_m_ value for myristoyl-CoA is much higher than that of acetyl-CoA. Thus, we measured the steady-state kinetic parameters for NMT1 catalyzed acetylation and myrisotylation of ARF6 peptide. Unexpectedly, the *k*_cat_ and *K*_m_ values were very similar (Figure 1 and Table 1).

**Table 1:** Steady-State Kinetic Parameters of NMT1^a^

substrate	*k*_cat_/*K*_CoA_, M^–1^min^–1^	*k*_cat_, min^–1^	*K*_CoA_^b^, μM	*K*_pep_^c^, μM	*K*_ia_, μM
Myr-CoA	(0.8 ± 0.1) × 10^6^	7.6 ± 0.6	9.8 ± 0.1	5.0 ± 0.8	0.2 ± 0.1
Ac-CoA	(1.3 ± 0.3) × 10^6^	2.7 ± 0.2	2.1 ± 0.5	40.0 ± 1.0	2.4 ± 0.5

a Enzymatic activity was measured at varying concentrations of acyl-CoA and ARF6 peptide in 50 mM TriCl at pH 8.0 and 37 °C.

b Michaelis constant *K*_m_ for acyl-CoA.

c Michaelis constant *K*_m_ for ARF6 peptide.

### NMT1 Prefers Myristoyl-CoA over Acetyl-CoA in Vitro Despite Similar *k*_cat_/*K*_m_ Values

If *k*_cat_/*K*_m_ values determine the substrate specificity, then NMT1 should be able to catalyze both acetylation and myristoylation of ARF6 in cells. However, we previously characterized ARF6 isolated from cells by MS, and did not find any acetylated ARF6.^5^ Thus, we reasoned that in this case, the *k*_cat_/*K*_m_ values were misleading.

**Figure 2:**
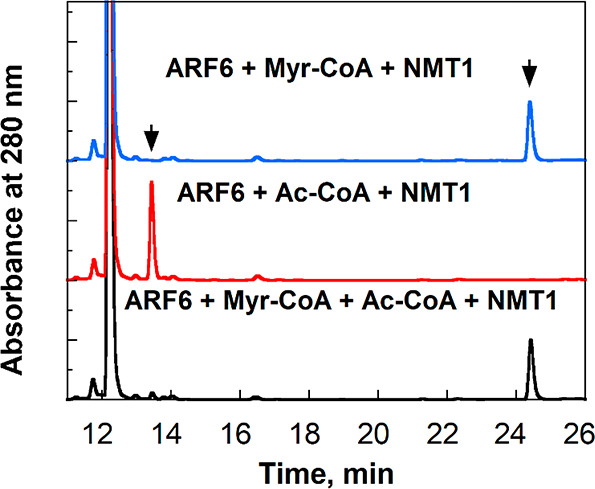
HPLC chromatograms of reactions of NMT1 with 200 μM ARF peptide, in the presence of 50 μM myristoyl CoA (blue), 50 μM acetyl CoA (red), or both 50 μM myristoyl-CoA and 50 μM acetyl-CoA (black). Reaction time was 1 h.

To further investigate this, we set up an NMT1 reaction with 200 μM ARF6 peptide and added both myristoyl-CoA and acetyl-CoA at 50 μM. In this reaction, ∼12 μM myristoylated ARF6 peptide was detected, similar to the reaction without acetyl-CoA (Figure 2). In contrast, acetylated ARF6 peptide witnessed a dramatic decrease from ∼15 μM in the reaction with acetyl-CoA only to barely detectable (∼1 μM) in the reaction with both acetyl-coA and myristoyl-CoA. Thus, NMT1 strongly prefers myristoyl-CoA over acetyl-CoA as the substrate despite the similar *k*_cat_/*K*_m_ values.

### Binding Affinities for Acyl-CoA Substrates Determines Substrate Specificity of NMT1

**Scheme 1:**
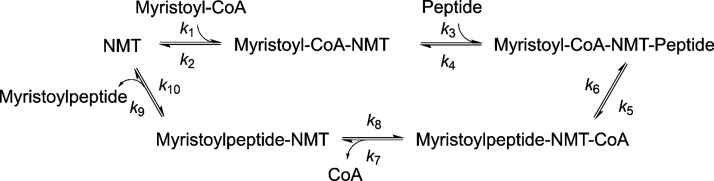
Minimal Mechanism of NMT

To explain why NMT1 almost exclusively uses myristoyl-CoA as a substrate when both myristoyl-CoA and acetyl-CoA are present even though the *k*_cat_/*K*_m_ values for them are similar, we hypothesized that NMT1 binds myristoyl-CoA much more tightly than it binds to acetyl-CoA, and thus, when both are present in the reaction, NMT1 will be almost completely occupied by myristoyl-CoA and catalyze only myristoylation. The initial evidence supporting this hypothesis comes from a smaller *K*_ia_ value of 0.2 ± 0.1 μM for myristoyl-CoA compared to 2.4 ± 0.5 μM for acetyl-CoA (Table 1). In an ordered Bi–Bi mechanism, *K*_ia_ represents the dissociation constant of the first substrate with free enzyme (= *k*_2_/*k*_1_ in Scheme 1). To test this hypothesis, we set out to measure the binding affinities of NMT1 toward myristoyl-CoA and acetyl-CoA. Efforts to directly measure the *K*_d_ values were unsuccessful because human NMT1 expressed and purified from *E. coli* already had myristoyl-CoA tightly bound. Incubation of 10 μM ARF6 peptide and 15 μM NMT1 without introduction of myristoyl-CoA led to the depletion of ARF6 peptide and accumulation of myristoylated ARF6 peptide (Figure S1), supporting the tight binding of myristoyl-CoA to NMT1. We recently published structures of NMT1 with myristoylated ARF6 peptide bound when we cocrystallized NMT1 with myristoyl-CoA and ARF6 peptide.^5^ However, we later noticed that electron density for the fatty acyl-CoA was observed in the crystals even when we did not add myristoyl-CoA, which again suggests that NMT1 binds myristoyl-CoA very tightly and the purified NMT1 already had myristoyl-CoA bound. Dialysis to remove the bound myristoyl-CoA was also not successful.

**Figure 3:**
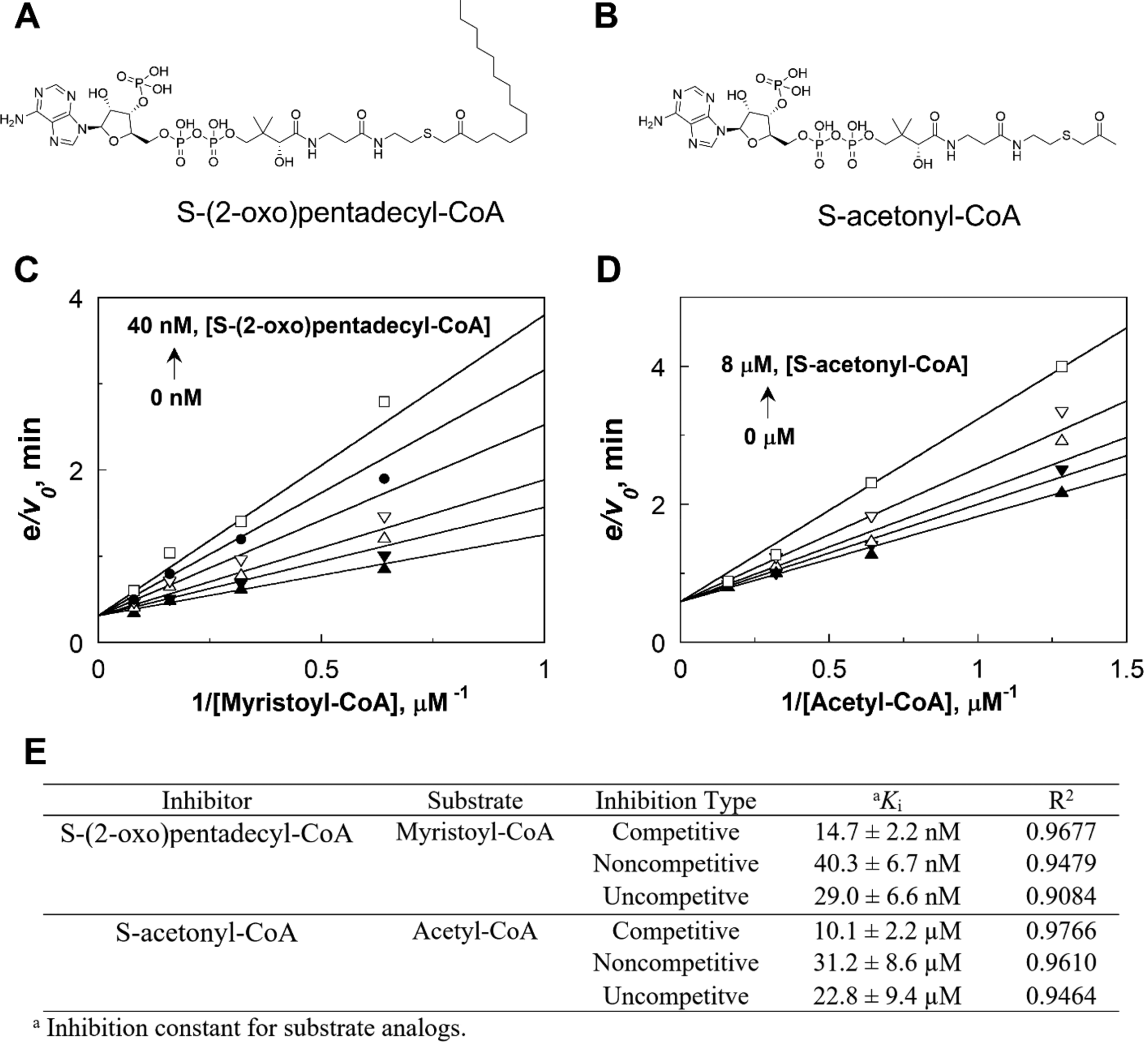
Graphic analysis of the inhibition of NMT1 by substrate analogues S-(2-oxo)pentadecyl-CoA and S-acetonyl-CoA. (A) Structure of myristoyl CoA analogue S-(2-oxo)pentadecyl-CoA. (B) Structure of acetyl-CoA analogue S-acetonyl-CoA. (C) Double reciprocal plot of the inhibition of NMT1 by S-(2-oxo)pentadecyl-CoA with myristoyl-CoA as the substrate. S-(2-oxo)pentadecyl-CoA concentrations were 0 (▲), 5 (▼), 10 (Δ), 20 (▽), 30 (●), and 40 (□) nM. (D) Double reciprocal plot of the inhibition of NMT1 by S-acetonyl-CoA with acetyl CoA as the substrate. S-acetonyl-CoA concentrations were 0 (▲), 1(▼), 2 (Δ), 4 (▽), and 8 (□) μM. (E) Inhibition constants of substrate analogues.

To better estimate the binding affinity between NMT1 and CoA substrates, we resorted to an enzyme kinetics method. We synthesized a myristoyl-CoA analogue, S-(2-oxo)pentadecyl-CoA, and an acetyl-CoA analogue, S-acetonyl-CoA (Figure 3 A and B). The introduction of a methylene bridge between the CoA sulfur and the acyl carbonyl makes the two analogues nonhydrolyzable. We then investigated the ability of S-(2-oxo)pentadecyl-CoA to inhibit the myristoyltransferase activity of NMT1 and the ability of S-acetonyl-CoA to inhibit the acetyltransferase activity of NMT1. In the myristoylation reactions, the concentrations of myristoyl-CoA and S-(2-oxo)pentadecyl-CoA were varied while the ARF6 peptide concentration was fixed at 12.5 μM, which is 2.5-fold higher than the *K*_m_. As shown in Figure 3C, a double reciprocal plot of the initial rates of reaction versus the concentration of myristoyl-CoA at fixed concentrations of S-(2-oxo)pentadecyl-CoA yielded lines intersecting on the *y*-axis, consistent with S-(2-oxo)pentadecyl-CoA being a competitive inhibitor of NMT1 against myristoyl-CoA. Likewise, in the acetylation reaction, the concentrations of acetyl-CoA and S-acetonyl-CoA were varied, and the ARF6 peptide concentration was fixed at 100 μM, which is 2.5-fold of the *K*_m_. S-Acetonyl-CoA was demonstrated as a competitive inhibitor of NMT1 against acetyl-CoA (Figure 3D). The initial rates were fitted to three classes of inhibition (e.g., competitive, noncompetitive, and uncompetitive). The best fit was obtained with eq 3 (see Material and Method in the Supporting Information), which describes a competitive inhibition pattern. The inhibition constants derived from the fit are summarized in Figure 3E. As competitive inhibitors compete with substrates for the enzyme active sites without the commitment to catalysis, inhibition constant *K*_i_ values represent the dissociation constant of the competitive inhibitors without the complication by catalytic steps as reflected in the *K*_m_ values of substrate. Considering the high structural similarity between the substrate analogues and substrates, the *K*_i_ values for S-(2-oxo)pentadecyl-CoA and S-acetonyl-CoA reflects the binding affinity of NMT1 for myristoyl-CoA and acetyl-CoA, respectively. The *K*_i_ value for S-(2-oxo)pentadecyl-CoA is determined to be 14.7 ± 2.2 nM (Figure 3E), close to the reported *K*_d_ value of ∼15 nM for yeast NMT with myristoyl-CoA.^8^ In comparison, the *K*_i_ value for S-acetonyl-CoA is 10.1 ± 2.2 μM, nearly 3 orders of magnitude larger than that of S-(2-oxo)pentadecyl-CoA, suggesting that acetyl-CoA likely binds with a *K*_d_ value of around 10 μM. This large difference in the estimated binding affinities with the two substrates explains the exceptional preference of NMT1 for myristoyl-CoA over acetyl-CoA, despite the fact that NMT1 has similar *k*_cat_/*K*_m_ values for them.

Previous studies established an ordered Bi–Bi reaction mechanism where acyl-CoA binds NMT prior to peptide and then acyl peptide release is followed by the dissociation of free CoA (Scheme 1).^9^ An alternative explanation for the substrate specificity of NMT1 could be the different interaction of ARF6 peptides with myristoyl-CoA-bound and acetyl-CoA-bound NMT1. The *K*_m_ for ARF6 peptide is 40.0 ± 0.5 μM with acetyl-CoA as the first substrate, which is 8 times higher than the *K*_m_ of 5.0 ± 0.8 μM with myristoyl-CoA as the first substrate. The smaller *K*_m_ value for ARF6 peptide with myristoyl-CoA indicates that NMT1 interacts with ARF6 peptide more efficiently toward catalysis after binding myristoyl-CoA than after binding acetyl-CoA, which is consistent with previous structural and kinetic studies demonstrating that myristoyl-CoA binding to NMT in a bent fashion leads to a conformational change allowing the binding of peptide substrate.^6,9,10^ The conformational change required to bind the peptide substrate might not occur as efficiently upon binding acetyl-CoA. This is further supported by the determination of *K*_CoA_ for myristoyl-CoA being almost 5 times larger than that for acetyl-CoA, which suggests that a significant fraction of the binding energy for myristoyl-CoA is not observed at the ground-state Michaelis complex but rather is used to drive a change in protein conformation that enhances the protein binding affinity for the ARF6 peptide. Despite the above analysis, the competition experiment in Figure 2 was done using the ARF6 peptide at a saturating concentration (200 μM), and thus, the differences in the *K*_m_ values for the ARF6 peptide could not explain the selectivity of NMT1 for myristoylation in the competition experiment.

### Binding Affinity Is Important for Determining the In Vivo Substrate Specificity of PTM Enzymes

The acyl-CoA specificity of human NMT demonstrates that the *k*_cat_/*K*_m_ value is not the best parameter to determine the in vivo substrate specificity of an enzyme. Instead, the binding affinities of substrates are more important for determining the substrate specificity of enzymes in a physiological setting. We found that this phenomenon is prevalent in biology. Enzymes that catalyze protein post-translational modifications often can coimmunoprecipitate with their substrate proteins. For example, SIRT1 and its deacetylation substrate p53 can coimmunoprecipitate,^11,12^ SIRT3 coimmunoprecipitates with its substrate IDH2,^13^ and furthermore, many of its substrate proteins can be coimmunoprecipitated and subsequently identified by mass spectrometry.^14^ The stress-activated protein kinase JNK coimmunoprecipitates with its upstream enzyme MEKK1^15^**and also its** substrate c-Jun.^16^ The transcription factor p53 and its E3 ubiquitin ligase can also be coimmunoprecipitated.^17^ The coimmunoprecipation of these enzyme–substrate pairs indicates that they have strong binding affinities.

According to the traditional view that *k*_cat_/*K*_m_ determines substrate specificity, an enzyme and its preferred substrate do not have to have a strong binding affinity.^18−20^ Instead, they just need to have higher *k*_cat_/*K*_m_ values. However, in cases where *K*_m_ = *K*_d_, *k*_cat_/*K*_m_ is still a valid specificity constant to use, we would like to emphasize that in the more complicated physiological conditions where multiple substrates with similar chemical and structural properties are present and compete for the enzyme active site, binding affinity should be more emphasized than *k*_cat_/*K*_m_ values. The fact that many enzyme–substrate pairs can coimmunoprecipitate strongly suggests that the determination of substrate specificity by binding affinity is prevalent in biology.

### Identification of Previously Unknown NMT1 Substrates by Searching for Its Binding Proteins

The appreciation that binding affinity is crucial for determining the substrate specificity of enzymes is of considerable importance for research that aims to understand the physiological function of PTM enzymes. To understand the functions of PTM enzymes, one important task is to identify what substrate proteins they modify. Many approaches have been developed to achieve this task. For example, phosphotyrosine antibodies are used to identify substrates of tyrosine kinases;^21^ bump-and-hole methods have been developed to identify protein kinase substrates;^22^ and protein methyltransferase substrates,^23^ pan acetyl-lysine, and pan succinyl-lysine antibodies were used to identify SIRT1 and SIRT5 substrates, respectively.^24,25^ However, in many cases, identifying the substrate proteins of a PTM enzyme remains a challenge for various reasons, such as the lack of proper affinity enrichment reagents or the low substrate abundance. The appreciation that binding affinity is more important than *k*_cat_/*K*_m_ values for determining substrate specificity in vivo can potentially provide a facile general or complementary approach to identify the substrate proteins of a PTM enzyme—instead of looking for proteins with the PTM, we can look for proteins that interact with the enzyme. Based on the understanding that a better substrate of the enzyme should also bind the enzyme more tightly, many of the interacting proteins should be the substrates of the enzyme, which can be biochemically validated. Because current proteomic technology is excellent at identifying interacting proteins, this will provide a facile solution to the challenge of PTM enzyme substrate identification.

To demonstrate the potential utility of this approach, we set out to use the existing interactome data for NMT1/2 to identify previously unknown NMT1/2 substrates. In the N-glycine myristoylation field, it is generally believed that the human substrate proteins are almost all identified due to the unique peptide sequence selectivity (NMTs prefer proteins with N-terminal GXXXS(K/R) sequences) of NMTs and various proteomic studies.^26,27^ Thus, the identification of previously unknown substrate proteins for human NMT1/2 would be a strong testament for the utility of this approach.

We therefore examined the NMT1 and NMT2 interacting proteins (Table S1) identified by the Gygi lab on the BioPlex Explorer.^28,29^ Out of 52 proteins that interact with NMT1 or NMT2, 19 (highlighted with green color in Table S1) are either known substrate proteins of NMT1/2 or have been identified as potential substrates in proteomic studies. This is a strong indication that NMT interactome studies could lead to the identification of NMT substrate proteins.

**Figure 4:**
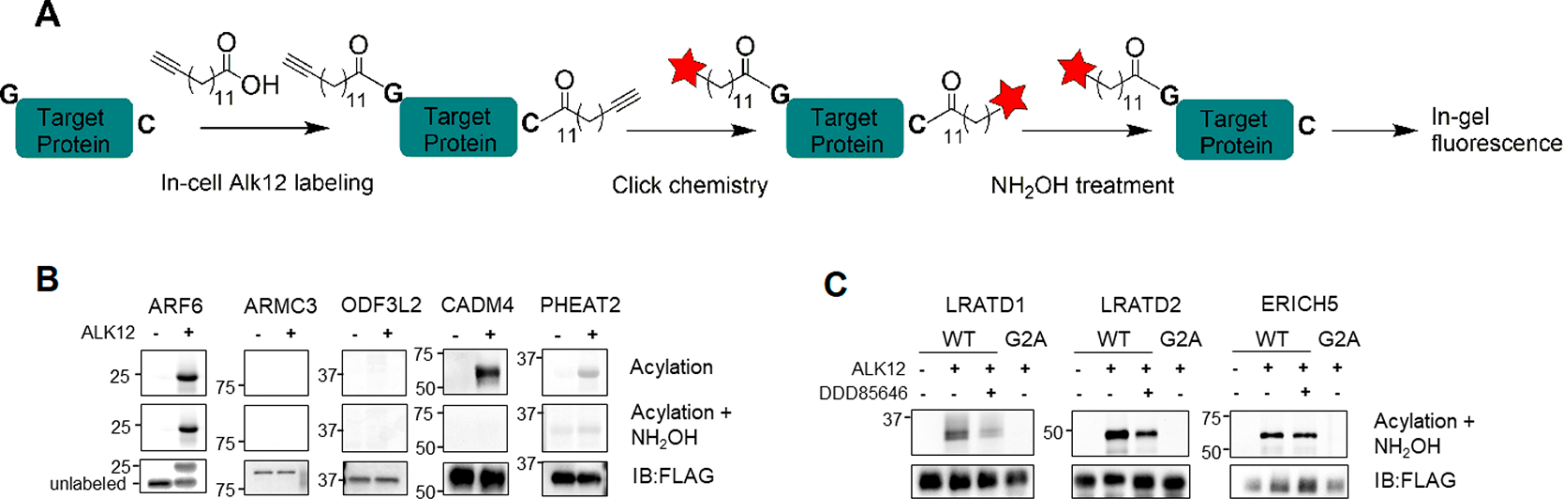
NMT substrate screening in cells. (A) Alk12 labeling flowchart. (B) Selected proteins in human NMT interactome were overexpressed in HEK293T cells with a C-terminal FLAG tag. Myristoylation level was monitored with Alk12 labeling with or without hydroxylamine. ARF6 served as the control. (C) LRATD1, LRATD2, and ERICH5 are NMT substrates as their acylation signals were decreased by NMT inhibitor and removed in G2A mutants.

To identify previously unknown substrate proteins from the interacting proteins, we focused our attention on the proteins that were not known to be NMT substrates. Among these, nine have N-terminal glycine or lysine (NMT could also myristoylate lysine side chain on N-terminal) residues, and we picked seven that we could obtain the expression vectors to biochemically validate them as NMT substrates. We transiently expressed them with C-terminal Flag tags in HEK293T cells and labeled with a clickable myristic acid alkyne (Alk12). Each protein was then purified with FLAG affinity pull down, and their fatty acylation levels were analyzed by in-gel fluorescence after TAMRA-azide conjugation (Figure 4A). Hydroxylamine was used to remove cysteine fatty acylation. ARF6 protein was used as a positive control in this experiment. As shown in Figure 4B, two proteins, ARMC3 (Armadillo repeat-containing protein 3) and ODF3L2 (outer dense fiber protein 3-like protein 2) did not show Alk12 labeling. Two other proteins, PHEAT2 (PH domain-containing endocytic trafficking adaptor 2, FAM109B) and CADM4 (cell adhesion molecule 4), showed Alk12 labeling signal but the signal was removed by hydroxylamine treatment, indicating that fatty acylation likely occurred on cysteine instead of N-terminal glycine. Thus, PHEAT2 and CADM4 are likely not substrates of NMT1/2. However, three proteins LRATD1 (LRAT domain-containing 1, also called FAM84A or neurological sensory protein 1 NSE1), LRATD2 (FAM84B/NSE2), and ERICH5 (glutamate-rich protein 5) demonstrated clear fluorescence signals in the Alk12 treated samples compared to the control without Alk12 treatment, and the signals were hydroxylamine-resistant, suggesting that they are potentially N-myristoylated. Further identification of LRATD1, LRATD2, and ERICH5 as NMT1/2 substrates was carried out with NMT inhibitor and G2A mutants. Mutating the N-terminal glycine to alanine (G2A) is known to prevent myristoylation by NMT1/2.^30^ Thus, if the G2A mutants are not labeled, it will further support that these proteins are N-terminally myristoylated by NMT1/2. Alk12 labeling signals decreased with the treatment of NMT inhibitor in LRATD1, LRATD2, and ERICH5 and were completely removed in G2A mutants, confirming LRATD1, LRATD2, and ERICH5 as substrates of NMT1/2 (Figure 4C).

Thus, by mining the available NMT1/2 interactome, we identified three previously unknown substrate proteins for NMT1/2. As mentioned, the N-glycine myristoylome was thought to be almost completely known. The identification of three previously unknown substrates of NMT1/2 demonstrates that the interactome analysis can be a powerful approach to identify the substrate proteins of PTM enzymes, especially given the readily available interactome data.

In conclusion, our study on NMT1’s preference for myristoyl-CoA over acetyl-CoA despite their similar *k*_cat_/*K*_m_ values led to the appreciation that *K*_d_ values are the key determining factor for the substrate specificity of an enzyme. While the NMT example may be a special case caused by *K*_m_ not equaling to *K*_d_, we propose that *K*_d_ should be more emphasized for determining the substrate specificity of enzyme, especially in a physiological setting where multiple substrate proteins compete for the same PTM enzyme. The enzymology field tends to emphasizes *k*_cat_/*K*_m_ values, which is easy to measure and useful for in vitro studies with a single substrate. Our study here indicates that for cellular and in vivo studies, where many substrate proteins compete for the same PTM enzyme, the binding affinity is very important and should be more emphasized. This understanding provides a clear rationale for the large body of literature showing that PTM enzymes and their substrate proteins can form stable complexes and be coimmunoprecipitated. The understanding will also have many practical applications as it allows the facile identification of substrate proteins for PTM enzymes, which will significantly speed up the understanding of the biological functions of these enzymes.

## Supporting Information Available

HPLC chromatogram of 1 h incubation ARF6 peptide with NMT1; steady-state parameter equations explaining NMT1 substrate preference; and material and methods (PDF)
Table S1 listing NMT1/2 interacting proteins from publicly available data (XLSX)
