# Supplementary material for: Binding Affinity Determines Substrate Specificity and Enables Discovery of Substrates for N-Myristoyltransferases
Source: ACS Catal. 2021 Nov 29;11(24):14877–83. doi: 10.1021/acscatal.1c03330 (PMC8689648; doi:10.1021/acscatal.1c03330)
Supplement: Supplementary file 1 — cs1c03330_si_001.pdf [file cs1c03330_si_001.pdf]

Supporting Information

Binding Affinity Determines Substrate Specificity and Enables Discovery of Substrates for N-Myristoyltransferases

Dan Su[1,3], Tatsiana Kosciuk[1,3], Min Yang[1], Ian R. Price[1], Hening Lin[1,2,*]

[1]Department of Chemistry and Chemical Biology, Cornell University, Ithaca, NY 14853, USA

[2]Howard Hughes Medical Institute; Department of Chemistry and Chemical Biology, Cornell University, Ithaca, NY 14853, USA

[3]These authors contributed equally.

*Corresponding author: hl379@cornell.edu

Supplementary Table S1: NMT1/2 interacting proteins from publicly available database. This is uploaded as a separate Excel file.

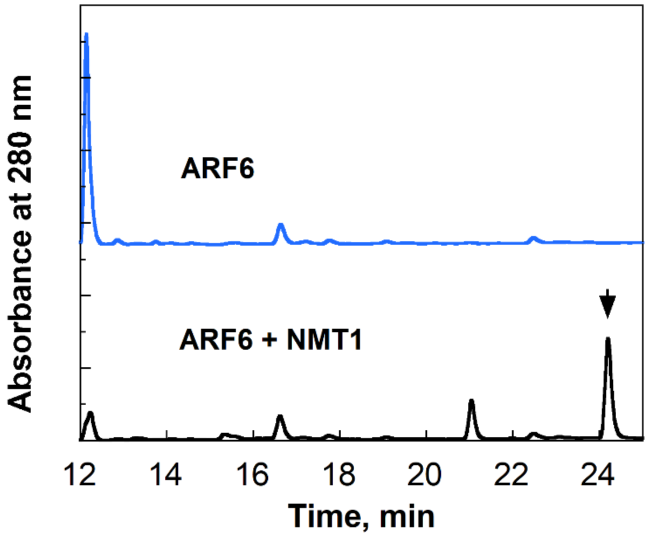

Figure S1. HPLC chromatogram of 1 h incubation of 10 µM ARF peptide, without (blue) and with (black) 15 µM NMT1. Arrow indicates the elution of myristoylated ARF peptide.

Why myristoyl-CoA and acetyl-CoA have similar k_{cat}/K_m values despite their different preference by NMT1. Previous studies established an ordered Bi-Bi reaction mechanism where acyl-CoA binds NMT prior to peptide, then acyl peptide release is followed by the dissociation of free CoA (see scheme below) (*J. Biol. Chem 266*:9732-9739, 1991) Michaelis constant for the acyl-CoA substrate (K_{CoA}) and k_{cat} were defined in Eq a and b (Segel H. I., in *Enzyme kinetics: behavior and analysis of rapid equilibrium and steady state enzyme systems*, Wiley, New York, 1975). k_{cat}/K_{CoA} for acyl-CoA thus equals k_1. The similar k_{cat}/K_{CoA} values for myristoyl-CoA and acetyl-CoA indicates two substrates bind to NMT1 with similar second order binding rate constant k_1. The large difference in binding affinity K_d, which is given by k_2/k_1, indicates that acetyl-CoA has a much larger dissociation rate k_2 compared to myristoyl-CoA. Thus, for myristoyl-CoA, k_3 (the forward chemical reaction rate) dominates while for acetyl-CoA, k_2 (the unbinding from NMT) dominates.

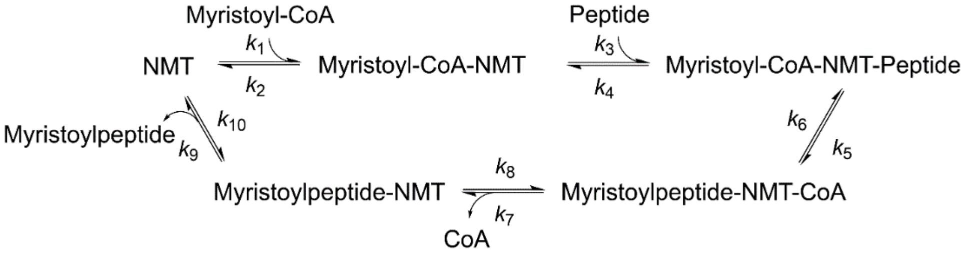

$$K_{CoA} = \frac{k_5 k_7 k_9}{k_1(k_7k_9+k_5k_7+k_5k_9+k_6k_9)} \quad \text{(a)}$$

$$k_{cat} = \frac{k_5 k_7 k_9}{k_7k_9+k_5k_7+k_5k_9+k_6k_9} \quad \text{(b)}$$

Material and Methods

Reagents. Anti-FLAG affinity gel (#A2220, RRID: AB_10063035), FLAG-HRP (#A8592, RRID: AB_439702, 1:5000 dilution), Tris[(1-benzyl-1H-1,2,3-triazol-4-yl)methyl]amine (TBTA), Tris(2-carboxyethyl)phosphine (TCEP), hydroxylamine, and protease inhibitor cocktail were from Sigma-Aldrich, 5-TAMRA azide from Lumiprobe (Hunt Valley, MD), Polyethylenimine (PEI) MAX transfection reagent (24765-1) from Polysciences (Warrington, PA), NMT inhibitor DDD85646 from Cayman Chemical (Ann Arbor, MI), ARF6 peptide from Biomatik (Wilmington, DE), Myristoyl-CoA and acetyl-CoA from Santa Cruz Biotechnology (Dallas, TX), and Pierce ECL Western Blotting substrate from Thermo Scientific (Rockford, IL). Alk12 was synthesized as previously reported.[26]

Plasmids. Human ARMC3, LRATD1, LRATD2, ERICH5, PHEAT2, CADM4 and ODF3L2 with C-terminal FLAG-tag in pcDNA3.1+/C-DYK plasmids were purchased from GenScript (Piscataway, NJ). G2A mutants of LRATD1, LRATD2, and ERICH5 were generated using site-directed mutagenesis.

Synthesis of S-(2-oxo)pentadecyl-CoA and S-acetonyl-CoA. To a solution of pentadecane-2-one (1.00 g, 4.40 mmol) in dry dichloromeththane (DCM, 20 ml) at 0 °C was added diisopropylethylamine (DIPEA, 1.08 mL, 6.20 mmol), 1,1,1,3,3,3-hexamethyldisiloxane (0.98 ml, 5.40 mmol). After stirring for 30 min, Na_2CO_3 (0.94 g, 8.8 mmol) and NBS (0.96 g, 5.40 mmol) were added. The resulting reaction mixture was warmed up to room temperature and stirred for two hours. Then 20 ml of sat. $NaHCO_3$ was added and stirred for 10 min and then allowed to settle. The organic lay was washed by water and brine and dried with anhydrous sodium sulfate. The solution was concentrated and the residue was purified by silica gel column chromatography (20:1 hexane:ethyl acetate) to afford 1-bromopentadecan-2-one (700 mg, 52.1% yield) as a white solid.

A solution of CoA trilithium salt (100 mg, 0.127 mmol), 1-bromopentadecan-2-one (188 mg, 0.618 mmol), DL-dithiothreitol (1.8 mg, 0.01mmol) in 40 mM Na_2CO_3 (10 mL) was stirred at room temperature for 16 hours. Then reaction was quenched by 1 ml of acetic acid. The reaction mixture was concentrated using a rotary evaporator and residue was purified by silica gel chromatography column (n-butanol:AcOH:H_2O=5:2:3) to afford S-(2-oxo)pentadecyl-CoA (86 mg, 68% yield) as a white solid. LC-MS, Calcd: 991.92; found: 992.54 (M+1), 1009.32 (M+H_2O), 1027.86 (M+2H_2O).

S-Acetonyl-CoA: At 0^0C, to a solution of CoA trilithium salt (50 mg, 0.064 mmol) and 1-chloropropan-2-one (7.6 μL, 0.095 mmol) in THF (3 mL) and water (3 mL) was added DIPEA (26.8 μL, 0.15 mmol). The reaction solution was warmed up to room temperature and stirred for 15 hours. Additional chloropropane-2-one (7.6 μL, 0.095mmol) was added and the reaction mixture as stirred for 5 more hours. The reaction was quenched by 50% aqueous acetic acid and concentrated using a rotary evaporator. The residue was purified by silica gel chromatography column (n-butanol:AcOH:H_2O=10:1:2) to afford S-Acetonyl-CoA (36 mg,67.9%) as white solid. LC-MS, Calcd: 823.60; found: 824.92 (M+1), 841.53 (M+H_2O)

Enzymatic assays. Enzymatic activity was measured by monitoring initial rates of acylated ARF peptide formation using an HPLC-based assay on a Shimazu UFLC. The steady-state kinetic parameters were obtained by varying concentrations of acyl-CoA and ARF peptide. For each 50 μL reaction, varied concentrations of acyl-CoA were first introduced in 44 μL 50 mM Tris-HCl, pH 8.0. Then 1 μL of ARF6 peptide of varied concentrations dissolved in DMSO was added and briefly vortexed. The reaction was initiated by adding 5 μL of 325 nM NMT1 in reaction buffer. The reactions were then briefly vortexed and incubated at 37 °C for 10 minutes in a heat block. Then the reactions were quenched with 50 μL acetonitrile and incubated at room temperature for

10 minutes. The samples were then centrifuged for 10 minutes at 20,000 × g, 4 ºC. The supernatants of 95 μL were transferred to borosilicate glass vials (Thermo Scientific/MSCERT5000-39TR) and 5 μL 50% trifluoroacetic acid (TFA) was added to each vial. The samples were analyzed using HPLC with a Kinetex 5u EVO C18 100A column (150 mm × 4.6 mm, Phenomenex). The gradient for HPLC analysis was 100% solvent A (water with 0.1% TFA) for 2 min, 0 to 35% solvent B (acetonitrile with 0.1% TFA) for 5 min, 35-64% solvent B for 15 min, 64-80% solvent B for 5 min, 89-100% solvent B for 2 min, 100% solvent B for 5 min and 100% solvent A for 5 min. To ensure rates were measured in steady state, the reaction is limited to ≤ 20% conversion of peptide substrate and the remaining concentrations of substrates after reaction were at least 10 times of the concentration of NMT1.

For the product detection by MS shown in Figure 1, the reactions were set up with the same method as mentioned above with 100 μM acyl-CoA, 100 μM ARF6 peptide and 32.5 nM NMT1. The reactions were incubated for 2 hours. The procedure for HPLC analysis was the same as described above. The LC-MS analysis of reaction products was conducted on Shimazu LC-20AD instrument coupled with a Thermo Scientific LCQ Fleet mass spectrometer. The supernatants collected after centrifugation was loaded on a Phenomenex Kinetex 5 μm EVO C18 column (50 mm × 2.1 mm) equilibrated with 100% buffer A (water with 0.1% acetic acid) and 0% buffer B (acetonitrile with 0.1% acetic acid). The elution gradient was 100% solvent A from for 3 min, 0-10% solvent B for 2 min, 10-100% solvent B for 5 min, 100% solvent B for 1 min and 0-100% A for 1 min. Substrates and products were monitored with electrospray in positive mode.

Reversible inhibition assays. Reversible inhibition of NMT1 by myristoyl-CoA analog S-(2-oxo)pentadecyl-CoA was carried out by varying both concentrations of myristoyl-CoA and S-(2-oxo)pentadecyl-CoA with 12.5 μM ARF6 peptide in 50 mM Tris-HCl, pH 8.0, by monitoring

the rate of myristoylated ARF6 peptide formation using HPLC. Reversible inhibition of NMT1 by acetyl-CoA analog S-acetonyl-CoA was investigated by varying both concentrations of acetyl-CoA and S-acetonyl-CoA with 100 μM ARF6 peptide in 50 mM Tris-HCl, pH 8.0, by monitoring the rate of acetylated ARF6 peptide formation using HPLC. The substrate analogs were dissolved in the reaction buffer and their concentrations were determined using a CoA extinction coefficient of 1.54×10^4 $M^{-1}cm^{-1}$at 260 nm. The reaction and HPLC procedures were the same as described above.

Data analysis. Kinetics data were fit with KaleidaGraph software (Synergy Software, Reading, PA). Apparent steady-state kinetic data were fit to Eq 1, the Michaelis-Menten equation, where S is the concentration of substrate acyl-CoA. Steady-state kinetic data were fit to Eq 2, which describes an ordered steady-state kinetic mechanism where v_o represents the initial velocity, e is the concentration of the enzyme, k_{cat} is the first-order macroscopic rate constant for enzyme turnover at saturating concentration of both acyl-CoA and ARF6 peptide, K_{CoA} and K_{pep} are the Michaelis constants for acyl-CoA and ARF6 peptide, respectively, and K_{ia} is the dissociation constant of the enzyme and acyl-CoA.

The kinetic data with substrate analog inhibitors were fit to Eq 3, 4 and 5, where S is the concentration of substrate acyl-CoA, I is the concentration of inhibitor and K_i is the dissociation constant for the inhibitor. Eq 3 describes a competitive inhibition pattern of the inhibitor versus acyl-CoA substrates. Eq 4 and 5 describe a non-competitive and uncompetitive pattern respectively.

$$\frac{v_o}{e} = \frac{k_{cat}S}{K_m + S} \tag{1}$$

$$\frac{v_o}{e} = \frac{k_{cat}\,[\text{Acyl-CoA}][\text{Peptide}]}{K_{CoA}[\text{Peptide}] + K_{pep}[\text{Acyl-CoA}] + [\text{Acyl-CoA}][\text{Peptide}] + K_{ia}K_{pep}} \tag{2}$$

$$\frac{v_o}{e} = \frac{k_{cat}S}{K_a\left(1 + \frac{I}{K_i}\right) + S} \tag{3}$$

$$\frac{v_o}{e} = \frac{k_{cat}S/\left(1+\frac{I}{K_i}\right)}{K_a+S} \tag{4}$$

$$\frac{v_o}{e} = \frac{k_{cat}S}{K_a+S\left(1+\frac{I}{K_i}\right)} \tag{5}$$

NMT substrate screening in vivo with Alk12 labeling. Human ARMC3, LRATD1, LRATD2, ERICH5, PHEAT2, CADM4, ODF3L2 and their mutants were transfected into HEK293T cells with polyethylenimine (PEI). After 24 hours, the cells were treated with 100 μM Alk12 with or without 2 μM NMT inhibitor DDD85646 and cultured for 24 hours before collection. Cells were lysed in NP-40 lysis buffer containing 25 mM Tris-HCl pH 7.4, 150 mM NaCl, 10% glycerol, and 1% Nonidet P-40 on ice for 30 minutes with brief vortex every 10 minutes. The lysates were then centrifuged at 17,000 × g for 10 minutes. The supernatants were collected and incubated with anti-FLAG affinity beads at 4 °C for 2 hours. The anti-FLAG affinity beads were then washed three times with IP buffer containing 25 mM Tris-HCl pH 7.4, 150 mM NaCl, and 0.2% Nonidet P-40 and resuspended in 20 μL of IP buffer. The click chemistry reactions were carried out by adding 1 μL of 2 mM 5-TAMRA dissolved in dimethyl sulfoxide, 1 μL of 10 mM TBTA dissolved in dimethylformamide (DMF), 1 μL of 40 mM $CuSO_4$ in water and 1 μL of 40 mM TCEP dissolved in water. The reactions were incubated in dark at room temperature for 1 hour. 10 μL of 2 × SDS protein loading dye was added and the mixtures were heated at 95 °C for 10 min before centrifugation at 17,000 × g for 2 minutes. The supernatants were collected and treated with 250 mM hydroxylamine at 95 °C for 7 minutes. 12 uL of each sample was loaded on a 12% polyacrylamide gel and allowed to be resolved at 220 V for 1 hour. In-gel fluorescence was detected with Typhoon FLA7000 (GE Healthcare Life Science). 3 μL of each sample was collected for western blotting analysis for protein loading.
